# Advances in genetic manipulation of *Chlamydia trachomatis*

**DOI:** 10.3389/fimmu.2023.1209879

**Published:** 2023-06-28

**Authors:** Weiqiang Wan, Danni Li, Dan Li, Jun Jiao

**Affiliations:** ^1^ State Key Laboratory of Pathogen and Biosecurity, Beijing Institute of Microbiology and Epidemiology, Beijing, China; ^2^ Department of Respiratory Medicine, Center for Pathogen Biology and Infectious Diseases, Key Laboratory of Organ Regeneration and Transplantation of the Ministry of Education, The First Hospital of Jilin University, Changchun, China

**Keywords:** intracellular bacterium, *Chlamydia trachomatis*, genetic manipulation, transformation, challenges

## Abstract

*Chlamydia trachomatis*, one species of *Chlamydia* spp., has the greatest impact on human health and is the main cause of bacterial sexually transmitted diseases and preventable blindness among all *Chamydia* spp. species. The obligate intracellular parasitism and unique biphasic developmental cycle of *C. trachomatis* are the main barriers for the development of tools of genetic manipulation. The past decade has witnessed significant gains in genetic manipulation of *C. trachomatis*, including chemical mutagenesis, group II intron-based targeted gene knockout, fluorescence-reported allelic exchange mutagenesis (FRAEM), CRISPR interference (CRISPRi) and the recently developed transposon mutagenesis. In this review, we discuss the current status of genetic manipulations of *C. trachomatis* and highlights new challenges in the nascent field of *Chlamydia* genetics.

## 1 Introduction

*Chlamydia* are a group of gram-negative, obligate intracellular pathogens, and their broad host ranges from single-celled eukaryotes to cattle, sheep and humans (1). The main species capable of commonly infecting humans include *Chlamydia trachomatis*, *Chlamydia pneumoniae*, and *Chlamydia psittaci*. Among them, *C. trachomatis* has the greatest impact on human health. The eye infection of *C. trachomatis* can cause trachoma, which is the main cause of preventable blindness in developing countries of the world (2). According to the data of the World Health Organization (WHO) in June 2022, at least 125 million people are living in trachoma-endemic areas face the risk of trachoma blindness (2). Besides, genitourinary infection caused by *C. trachomatis* may lead to venereal lymphogranuloma or infertility (3).

*C. trachomatis* has a unique biphasic developmental cycle that alternates between two morphologically and functionally distinct developmental stages: the small, structurally stable, infectious elementary body (EB) and the large, metabolically vigorous, replicative reticulate body (RB) (1, 4). Infection begins with attachment and internalization of EBs to host cells. RBs replicate within a membrane bound compartment - the inclusion, early genes are transcribed and EBs differentiate into RBs (~6–8 hours post-infection) (1, 5, 6). Next, effectors that mediate nutrient acquisition and maintain the viability of the host cell are expressed. The bacteria divide by binary fission and the inclusion substantially expands (~8–16 hours post-infection) (6, 7). Last, RBs gradually re-differentiate back into EBs. By host cell lysis or by extrusion of intact inclusions, EBs are released to infect neighboring host cells at the end of the developmental cycle (~24–72 hours post-infection) (1, 8, 9).

Although the sequence of the first *C. trachomatis* genome was published in 1998 (10), the functional analysis of proteins of *C. trachomatis* has been limited by the lack of tools of genetic manipulation for a long time. A major barrier to the development of genetic manipulation is its dependence on a host replication and the unique biphasic developmental cycle. RB is only present in the host cell, and it is difficult for exogenous DNA to reach the bacterial cytoplasm through the four-layers of biofilm (11). Even EB can exist in the environment, it has a hard cell wall and low metabolic activity, so it is unlikely to reabsorb and integrate foreign DNA (11).

**Figure 1:**
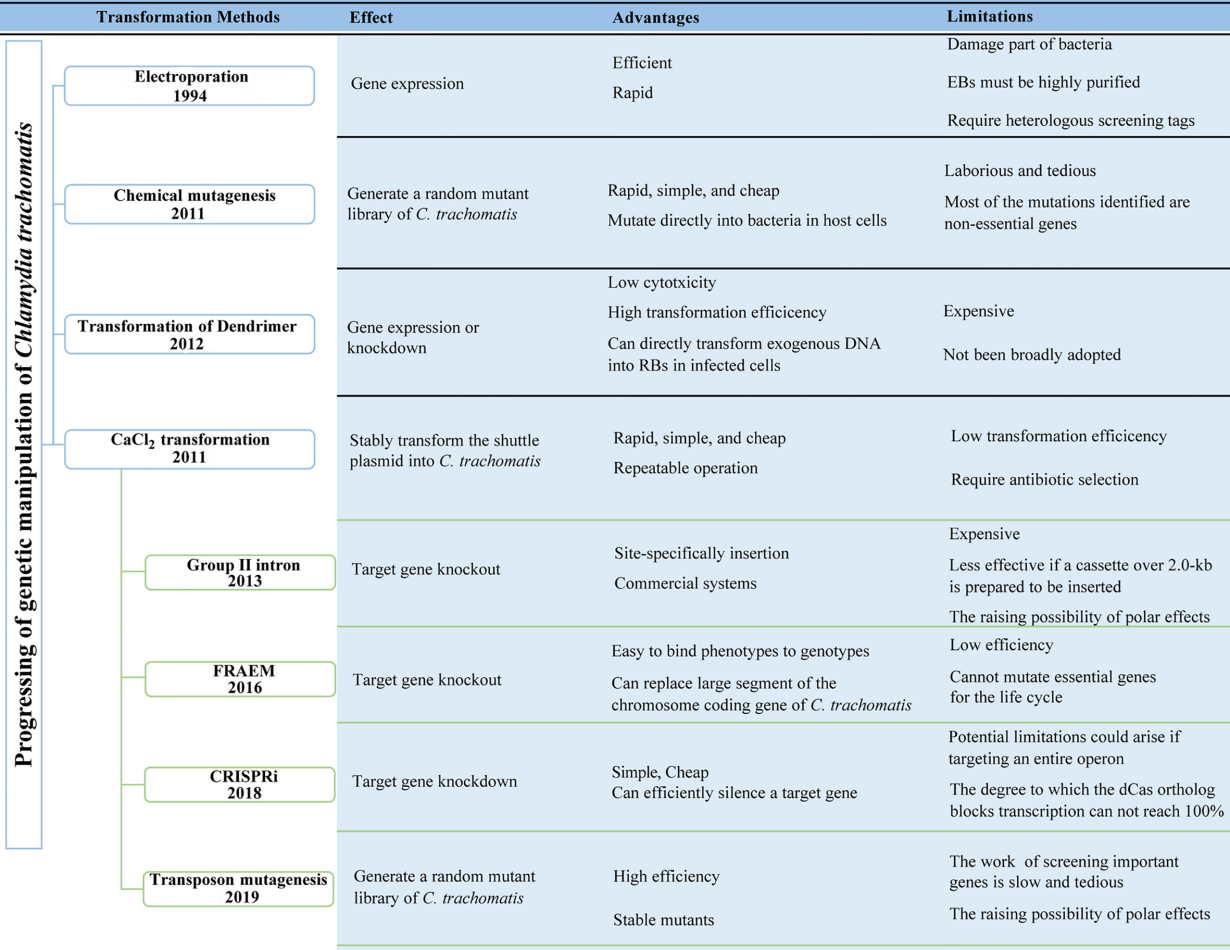
Progressing of the genetic manipulation of *Chlamydia trachomatis*. FRAEM, fluorescence-reported allelic exchange mutagenesis. CRISPRi, CRISPR interference.

Fortunately, in the past decade, the development and application of several tools of genetic manipulation of *C. trachomatis* has made some progress, greatly expanding the current research on the biological characteristics of *C. trachomatis* and the function analysis of its virulence factors. The first step in almost all methods of genetic manipulation of *Chlamydia* is transformation. Four transformation methods have been reported: electroporation, chemically induced mutagenesis, polyamidoamine dendrimers (PAMAM dendrimers), and CaCl_2_ transformation (4, 12, 13). In this review, we categorize and summarize the tools of genetic manipulation that have been developed for *C. trachomatis* according to the methods of plasmid transformation (**Figure 1**; **Table 1**).

**Table 1:** Progressing of the genetic manipulation of *Chlamydia trachomatis*.

Transformation methods		Firstly reported	Procedures	Selectable markers	Transformation achievements	References
Electroporation	\	Tam et al. 1994	1. Highly purified EBs 2. Brief electric pulses 3. Antibiotic selection	Chloramphenicol	Expression of chloramphenicol resistance by transformation with a shuttle plasmid	(14)
Chemical mutagenesis	\	Kari et al. 2011	1. *Chlamydia* infection 2. Exposure to either of EMS 3. Plaque isolation	**\**	Generate a random mutant library of *Chlamydia trachomatis*	(15–18)
Transformation of dendrimer	\	Mishra et al. 2012	1. *Chlamydia* infection 2. Dendrimer-plasmid complexes preparation 3. Antibiotic selection	\	The efficient and highly specific knockdown of transcript levels from targeted genes	(19, 20)
CaCl_2_ transformation	Group II intron-based targeted gene knockout	Johnson et al. 2013	1. Crude purified EBs 2. Targeting the intron by TargeTron system 3. Transformation and creation of site-specific, insertionally-inactivated mutants 4. Antibiotic selection 5. Genotyping analyses of mutants	Ampicillin	Group II introns can be retargeted by altering DNA sequences within the intron’s substrate recognition region to create site-specific gene insertions to achievetarget target gene knockout	(21–30)
	Fluorescence-reported allelic exchange mutagenesis (FRAEM)	Mueller et al. 2016	1. Crude purified EBs 2. Plasmid construction 3. Transformation and FRAEM 4. Antibiotic selection 5. Validation and sequencing	Green fluorescent protein and penicillin	Creation of GFP-expressing bacteria *via* homologous recombination between wild-type gene on a suicide plasmid containing *gfp* and it on the chromosome to achievetarget target gene knockout	(31–38)
	CRISPR interference (CRISPRi)	Ouellette et al. 2018	1. Crude purified EBs 2. Plasmid construction and transformation 3. Antibiotic selection 4. aTc was added or not to induce expression of the dCas9 5. Analysis	Penicillin	Inducibly and reversibly repress gene expression in *C. trachomatis* to achieve target gene knockdown	(39–43)
	Transposon insertion mutagenesis	LaBrie et al. 2019	1. Crude purified EBs 2. Plasmid construction and transformation 3. Antibiotic selection of the transposon mutants 4. Isolation of individual mutants 5. Assembly and analysis of the transposon mutant genomes	Ampicillin	Generate a single transposon-insertion mutant clones of *C. trachomatis*	(44–46)

## 2 Electroporation

Electroporation is universally effective in introducing heterologous DNA into obligate intracellular bacteria *via* brief electric pulses, which induce transient and reversible cell membrane permeabilization (47), and time constant and exponential decay pulse types, episomal DNA maintenance are the key features. Tam et al. successfully introduced the shuttle plasmin pPBW100 into *C. trachomatis* EBs by electroporation for the first time in 1994, and used it to infect McCoy cells and detected *Chlamydia* containing the chloramphenicol resistance gene in cell cultures (14). A similar electroporation method was used to mobilize an engineered vector into *C. psittaci* 6BC EBs and a efficiency was obtained with 10 µg of circular vector (1.9 ± 1.1×10^6^, n=7) (48). For the successfully transformation, EBs must be highly purified and obtained by centrifugation through Renografin density gradients, and this complex procedure maybe limit the wide adoption of electroporation by other labs. Although the optimal transformation conditions for *C. trachomatis* electroporation have not been fully grasped, the study of Tam shows that exogenous DNA can be introduced into EBs by electroporation and the expression of heterologous screening tags can be achieve, which laid the foundation for the development of *C. trachomatis* electroporation technology in the future (14).

## 3 Chemical mutagenesis

Chemical mutagenesis is a technology that uses chemical mutagens such as base analogues, deamination agents, and alkylating agents to mutate DNA (49, 50).

*C. trachomatis* was subjected to low-level ethyl methanesulfonate (EMS) mutagenesis to generate *chlamydiae* that contained less than one mutation per genome in 2011 and a tryptophan synthase gene (trpB) null mutant incapable of avoiding the anti-microbial effect of IFN-γ–induced tryptophan starvation was isolated (15). Then mutagenesis in *Chlamydia* was performed by exposure of infected cells to either of the DNA alkylating compounds EMS or N-ethyl-N-nitrosourea (ENU), followed by plaque isolation of clonal strains in 2015 (4). In the study, Kokes et al. used ethyl methyl sulfonate (EMS) and N-ethyl-N-nitrosourea (ENU) to perform chemical mutagenesis on *C. trachomatis* to generate a mutant library, screening of mutants impaired in F-actin assembly and identifying InaC as an inclusion body membrane protein that binds host ARF and 14-3-3 proteins and regulates F-actin recombination and Golgi reorganization around vesicles (16).

Although studies have shown that it is possible to achieve either one mutation (15) or multiple mutations per genome of *C. trachomatis* (16–18) by adjusting different concentrations of mutagen, identifying and linking genotype and phenotype without a molecular signature is laborious and tedious, and most of the mutations identified are non-essential genes (51). Therefore, with the continuous development of genetic manipulation, the application of chemical mutagenesis will be gradually phased out.

## 4 Transformation of dendrimer

Polyamidoamine (PAMAM) dendrimers are hyperbranched polymers with low cytotoxicity. It can not only deliver small molecules to specific sites, but also effectively transfuse biological macromolecules such as oligonucleotides and plasmid DNA into cells (52). In addition, these dendrimers can be localized in *Chlamydia* inclusion bodies in *Chlamydia*-infected cells (53), indicating that PAMAM can directly transform exogenous DNA into RBs in infected monolayers (19, 52).

In 2012, Mishra et al. used PAMAM to transfer an antisense oligonucleotide into *C. trachomatis* and efficiently and specifically knockdown the transcription level of the target gene (19). Then in 2013, Kannan successfully used PAMAM to transfer a plasmid (pMW82: pL2-01-pL2-01P-GFP) into *C. trachomatis* and successfully detected green fluorescence in the initial transformed culture (20, 48). Despite plasmid replication and GFP expression being detected within the first infection cycle which indicating a high transformation efficiency, the dendrimer-based transformation method of *Chlamydia* has not been broadly adopted.

## 5 CaCl_2_ transformation

Since the first utilization of CaCl_2_ transformation method to stably transform the shuttle plasmid pBR325::L2 into *C. trachomatis* EBs by Wang et al. in 2011 (54). In the protocol, EBs were firstly incubated with plasmid DNA in CaCl_2_ buffer for 30 min at room temperature and then host cells resuspended in CaCl_2_ buffer were added, followed by an additional incubation for 20 min at room temperature. Due to its advantages of simple, rapid, cheap and repeatable operation, the CaCl_2_ transformation method is widely used as a general transformation method for *Chlamydia*. For the CaCl_2_ transformation, crude preparations of *Chlamydia* from host cell lysates exhibit more efficient than gradient purified EB preparations (54–56). Based on this method, group II intron-based targeted gene knockout (21–30), fluorescence-reported allelic exchange mutagenesis (FRAEM) (31–38), CRISPR interference (CRISPRi) (39–43) and transposon insertion mutagenesis (44–46) have been realized in *C. trachomatis*.

### 5.1 Group II intron-based targeted gene knockout

Group II introns are a class of self-splicing ribozymes capable of high-frequency movement between genes through a retrohoming (TargeTron system) with the help of intron-encoded protein (IEP, with RAN maturase, endonuclease, and reverse transcriptase activities) (21, 57). Based on this principle, the first targeted disruption of a gene on *Chlamydia* chromosome was performed by Johnson and Fisher in 2013 (21). In the study, a plasmid containing the coding sequence of β-lactamase was transformed successfully and site-specifically, insertionally inactivated *incA* of *C. trachomatis* L2 strain, confirming the requirement of this protein for homotypic fusion of Chlamydial inclusion (21, 25, 58).

Right now, group II intron integration technology has been used in commercial systems such as Sigma’s TargeTron gene knockout system, and it has been successfully used for gene knockout of other intracellular parasitic bacteria including *Ehrlichia* and *Rickettsia* (21, 59). However, this method requires bioinformatics analysis to determine the intron insertion site, so it is necessary to design several insertion sites at the same time to ensure the probability of gene knockout (21, 57, 60, 61), and it is also less effective if a cassette over 2.0-kb is prepared to be inserted. Another major limitation of this system is that intron insertions may have polar effects on the expression of neighboring genes if the knocked-out chlamydial genes exist within polycistronic operons.

### 5.2 FRAEM

In 2013, Wickstrum et al. developed an inducible gene expression system (shuttle plasmid pASK-GFP-L2) for *Chlamydia*, in which gene expression was controlled by Tet, developing a strategy for gene expression and/or complementation (62). Song et al. reported that pgp6 on the native pL2 plasmid of *C. trachomatis* is necessary for this plasmid maintenance (63). Then in 2016, Mueller et al. constructed a suicide plasmid pUS6 based on the inducible expression of pgp6 and permit rapid reverse genetics by FRAEM (31). This system can replace the chromosome coding gene of *C. trachomatis* with a 2.2-kb cassette encoding both GFP and β-lactamase, thus realizing the targeted knockout of *C. trachomatis* gene and permitting the monitoring of mutagenesis by fluorescence microscopy. They successfully constructed the *trpA*-deficient strain of *C. trachomatis* and found that the deficient strain was unable to grow in indole-containing medium (31). Later, they adapted FRAEM technology by leveraging a step-wise Cre-lox approach to excise selection marker genes from a deleted gene locus to eliminate the possibility of polar effects mediated by the inserted cassette (33).

Recently, Kenneth et al. present functional evidence that the region between *C. trachomatis* pgp6 and pgp7, containing four 22-bp tandem repeats in the endogenous plasmids, represents the chlamydial native plasmid origin of replication (32, 64). They constructed plasmid pKW-L2ori by mobilization of the entire region between these two genes from chlamydial native plasmid pL2 into a pUC19-based plasmid and proved that it could be maintained by *C. trachomatis* serovar D which contains a native chlamydial plasmid. Subsequently they proved that pKW can be utilized as a conditionally replicating plasmid sufficient for the generation of deletion mutants *via* allelic exchange (32).

Although FRAEM can specifically knockout gene of *C. trachomatis*, this strategy requires a low-frequency double-crossover event. Further optimized methods including using some heterologous site-specific recombinases [which have been reconstructed and applied to *Coxiella burnetii* (65)] could be applied to assist gene recombination in *C. trachomatis*.

### 5.3 CRISPRi

Since its release in 2012, the CRISPR/Cas9 system has been widely used due to its simple operation, low cost, and high efficiency (66). To repurpose the CRISPR system for transcription regulation, Matthew et al. have described an RNA-based method, CRISPR interference (CRISPRi), and they have shown that CRISPRi can efficiently silence a target gene with up to 99.9% in *Escherichia coli* (67). Until now, CRISPRi has been used for targeted silencing of transcription in intracellular bacteria including *Mycobacterium tuberculosis* (68), *C. burnetii* (69, 70).

In 2018, Ouellette successfully knocked down *incA* gene of *C. trachomatis* by using CRISPRi based on the catalytically inactive Cas9 variant (dCas9) of *Staphylococcus aureus*, proving that the system can be used to reversibly inhibit *incA* expression, in addition that they found the plasmid encoding the dCas9 from *Staphylococcus pyogenes* was not possible to successfully transform *C. trachomatis* with it (42). And in 2021, Ouellette et al. optimized and improved the missing expression of anhydrotetracycline (aTc) - inducible dCas9 orthologous genes and plasmid instability in the original system, and developed a second CRISPRi system based on the dCas12 system to expand the number of potential chromosomal targets (41). These two CRISPRi systems will allow for broad targeting of the *C. trachomatis* genome and for analysis of essential gene functions in *C. trachomatis* in a straightforward manner.

However, Wurihan et al. successfully transformed two plasmid encoding staphylococcal (*S. aureus and S. pyogenes*) dCas9 to *C. trachomatis* and found that conditional expression of the staphylococcal dCas9 strongly inhibits chlamydial growth in the absence of any specific guide RNA (gRNA) (40), suggesting that the staphylococcal dCas9 proteins in their current forms have limited utility for chlamydial research and strategies to overcome this problem should be developed.

### 5.4 Transposon mutagenesis

Transposon mutagenesis is an effective method for discovering specific genetic components associated with a given phenotype. The basic principle is that when a transposase drives an exogenous transposon integrating into the promoter region or coding region of an unknown gene randomly, the gene will be inactivated and a new mutant phenotype will be produced. Transposon mutagenesis has been applied to *C. burnetii* (71, 72), *Rickettsia felis* (73), *Rickettsia prowazekii* (74) and *Ehrlichia chaffeensis* (75) for the identification of virulence proteins.

In 2019, LaBrie et al. constructed a non-replicating plasmid termed pCMA to encode the widely utilized C9 Himar1 transposase (44). The pCMA plasmid was used in a *C. trachomatis* transformation procedure with β-lactams for selection and then a pool of 105 transposon mutant clones from 23 transformations was generated. Further experiments proved that a FAD-dependent monooxygenase (*ct148*) and a deubiquitinase (*ct868*) were important for infection, and identified CT339 as a ComEC (the DNA-uptake protein) homolog important for DNA uptake and lateral gene transfer (44). O’Neill et al. then describe the first application of a Transposon Directed Insertion Site sequencing (TraDIS) - based approach to *C. trachomatis*, offering a novel approach for saturation mutagenesis and thus identifying gene essentiality/functionality (45, 46). Later, transposon mutagenesis of *Chlamydia muridarum* was also development and 33 transposon mutants were generated from a total of 10 independent transformation experiments (76).

The development of transposon mutagenesis in *C. trachomatis* provides additional avenues for discovering the molecular mechanism underlying the pathogenesis of *C. trachomatis* and for a more thorough understanding of this important pathogen (44). A limitation of transposon mutagenesis is the raising possibility of polar effects mediated by the inserted transposon due to polycistronic operons existing within the chlamydial genes.

## 6 Summary and prospectives

The genetic intractability of *C. trachomatis* has severely limited molecular dissection of virulence factors associated with intracellular parasitism and pathogenic mechanisms that promote trachoma, venereal lymphogranuloma or infertility, because there was no methods for *C. trachomatis* virulence determinants inactivation and/or complementation. Great progress has been made in the development of genetic manipulation of *Chlamydia* in the past decade, and the application of the tools of genetic manipulation has significantly impeded progress in understanding the genetic basis of the pathogen’s unique lifestyle and virulence. Moreover, the increasing genetic tractability of *C. trachomatis* will enable the development of new pathogen countermeasures, such as rationally designed attenuated or subunit vaccines. But some problems still remain:

(1) Low transformation efficiencies remain an obstacle to further development of genetic tools. Reasons including suboptimal electroporation conditions/buffers, purity of host cell-derived organisms could be account for the poor efficiency.
(2) At present, the developed tools of genetic manipulation are mostly suitable for *C. trachomatis* and incapable for the commonly infecting humans pathogens include *C. pneumoniae* and *C. psittaci*.

Additional advances in genetic manipulation will be necessary to render *Chlamydia* significantly more genetically tractable. Ideally, a cell-free medium like *C. burnetii* (77, 78) for *Chlamydia* cultivation would address some issues. Omsland et al. developed a stage-specific metabolic and transcriptional activity of *C. trachomatis* in an axenic medium in 2012 (79), and host-free cultivation of *Chlamydia* may be achievable in the future. Improved electroporation conditions may be another avenue if the decreased chlamydial viability could be addressed. Overall, more rapid and definitive progress can be expected for this important and interesting intracellular parasite.

## Author contributions

The manuscript was drafted by WW and DannL, and edited by JJ and DanL. All authors contributed to the article and approved the submitted version.
